# Supplementary material for: Population density and basic reproductive number of COVID-19 across United States counties
Source: PLoS One. 2021 Apr 21;16(4):e0249271. doi: 10.1371/journal.pone.0249271 (PMC8059825; doi:10.1371/journal.pone.0249271)

**S1 Appendix. Population density and basic reproductive number of COVID-19 across United States counties – Supporting Information.**

**Authors:** Karla Therese L. Sy, Laura F. White, Brooke E. Nichols

**Table of Contents:**

**S1 Appendix Fig A.** Period of calibrated exponential growth of cases in US counties (n=1,151).

**S1 Appendix Fig B.** Period of calibrated exponential growth of deaths in US counties (n=301).

**S1 Appendix Fig A.** Period of calibrated exponential growth of cases in US counties (n=1,151).

The plots of the log of cumulative cases over time for each county demonstrated reasonable curves that approximated exponential growth. The calibrated exponential growth period was used to estimate R_0_.


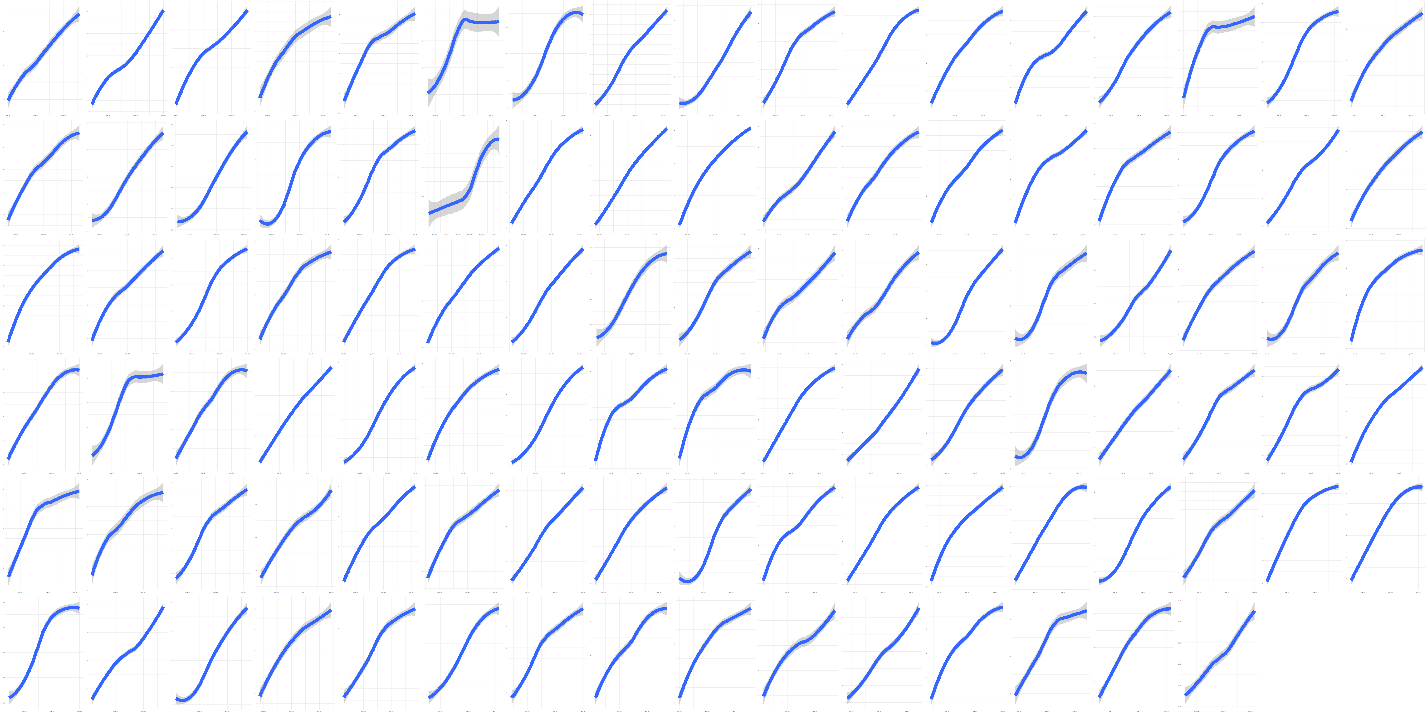

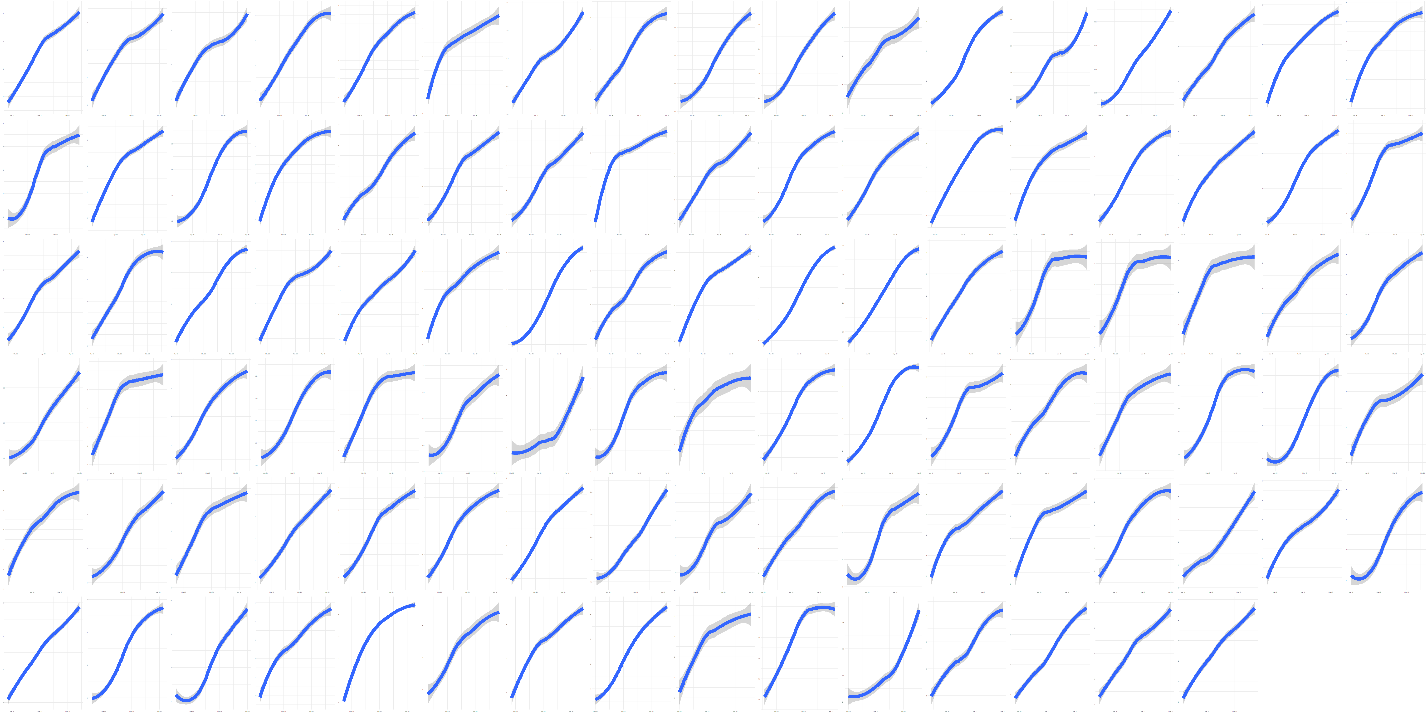

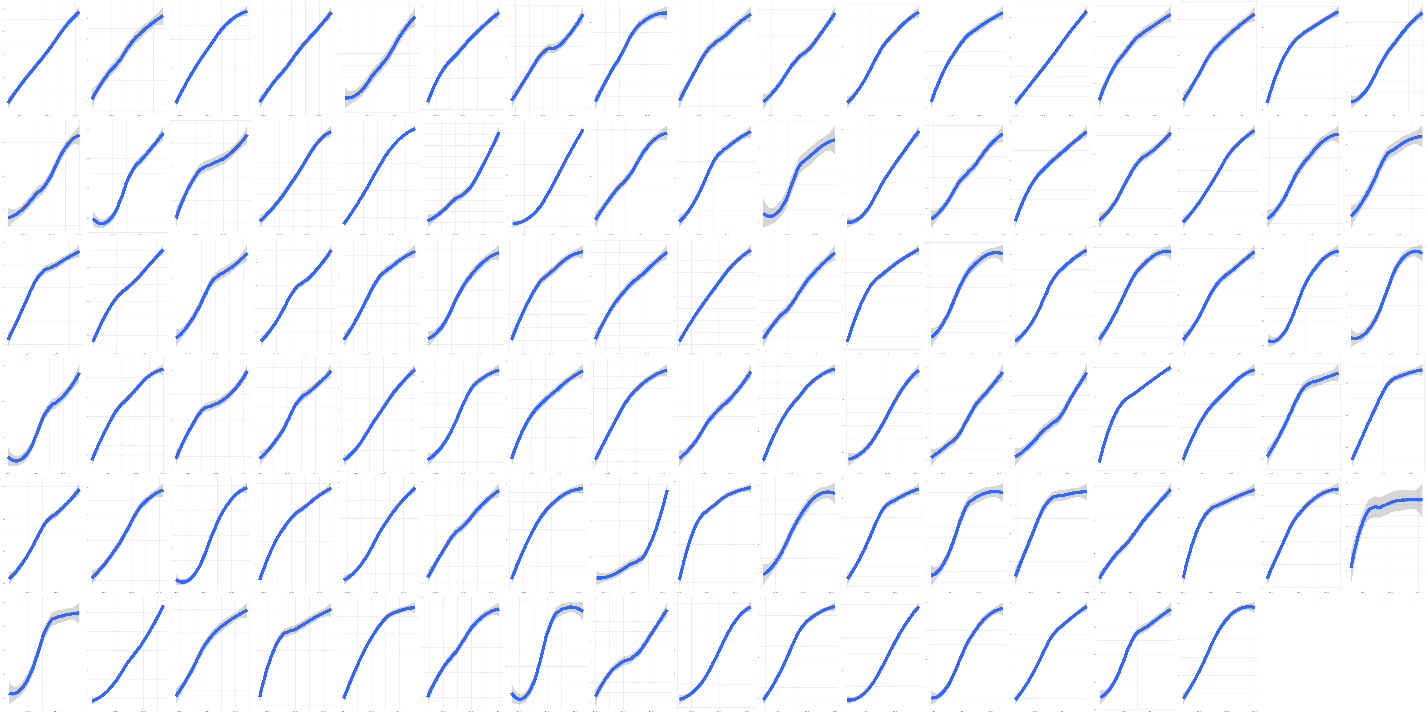

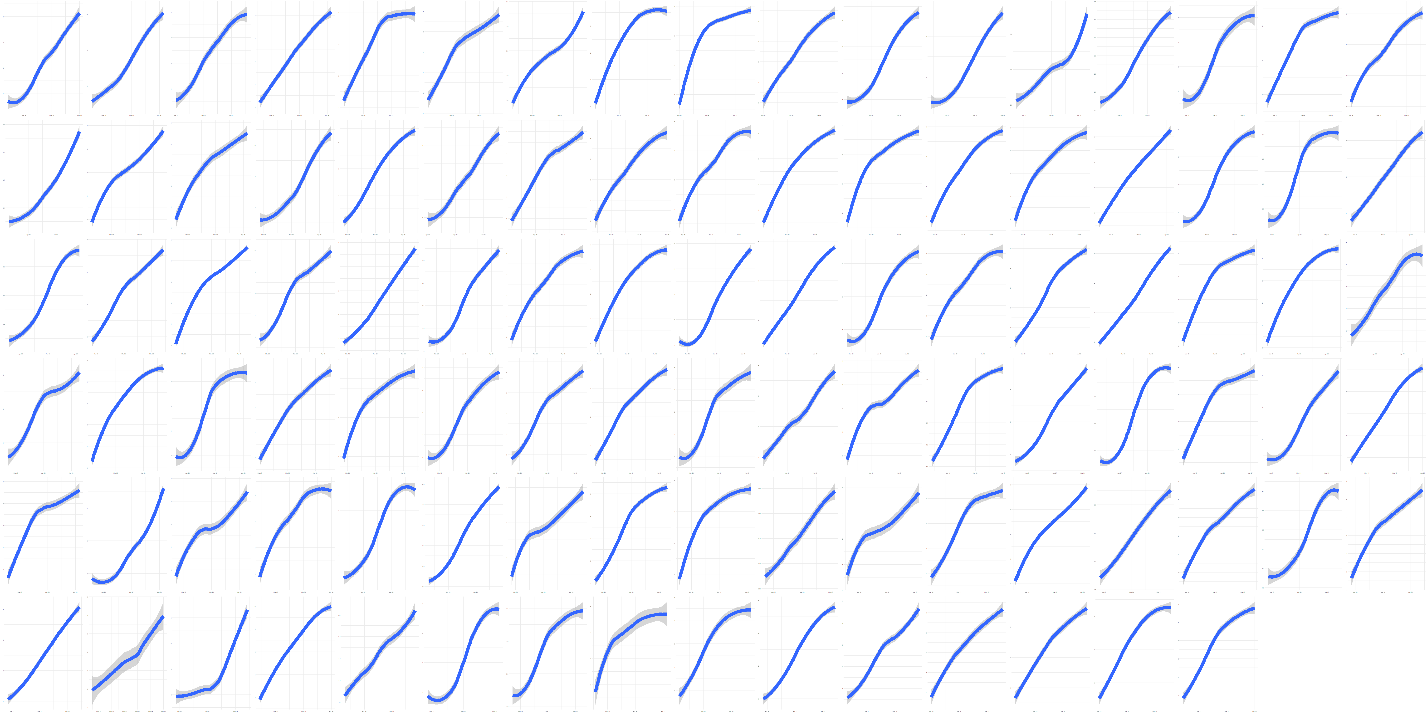

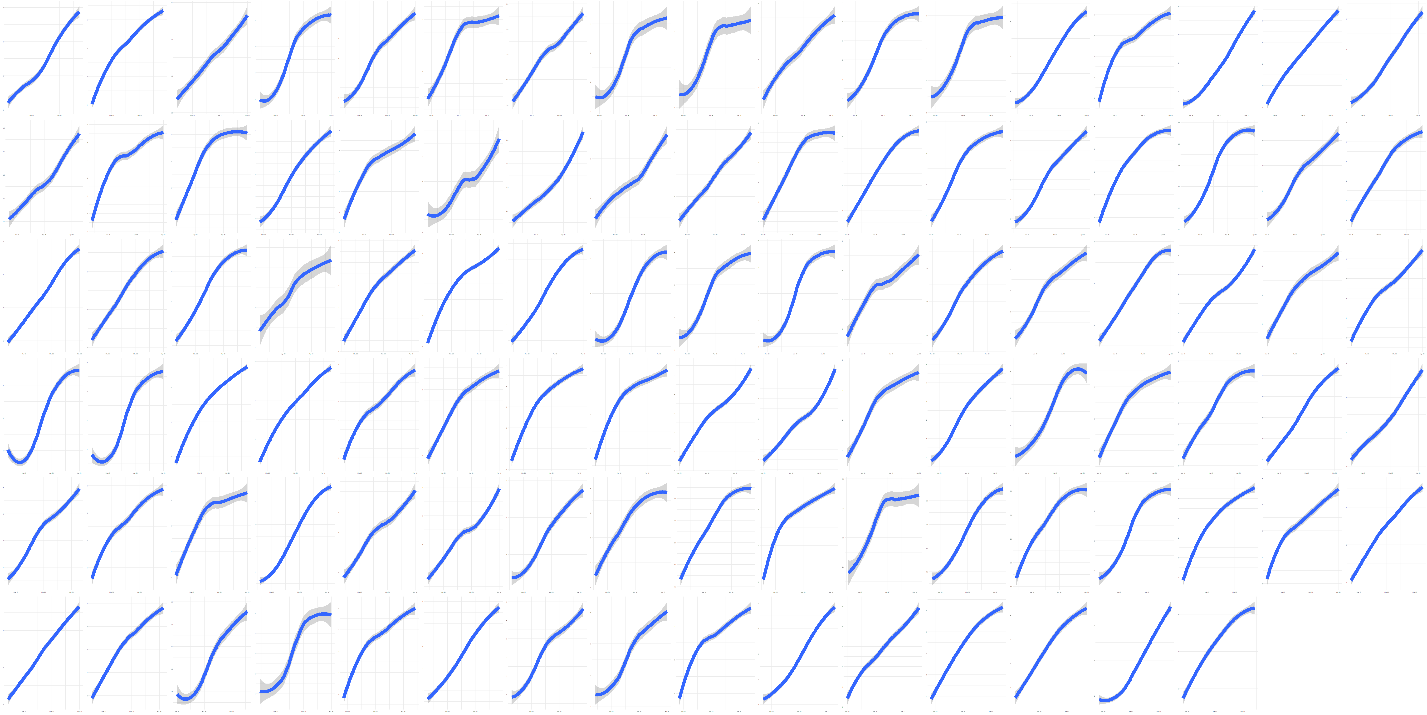

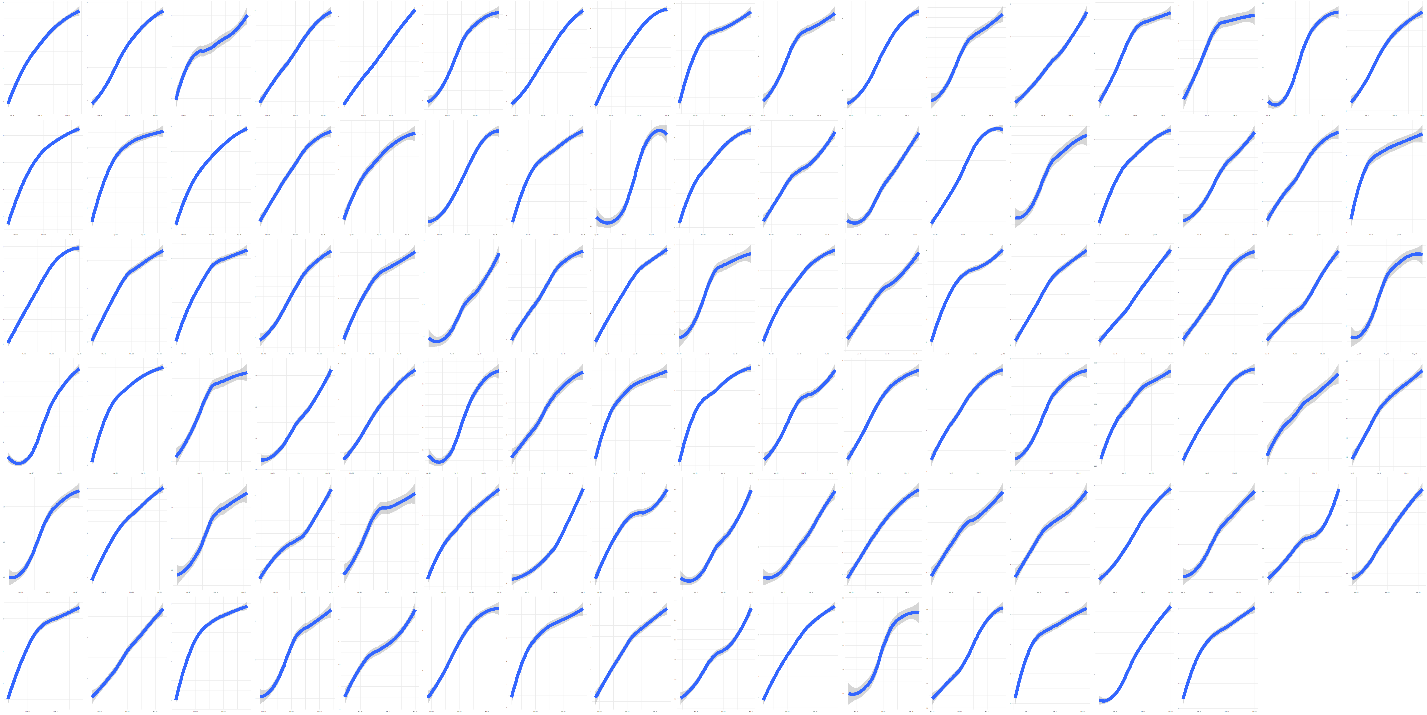

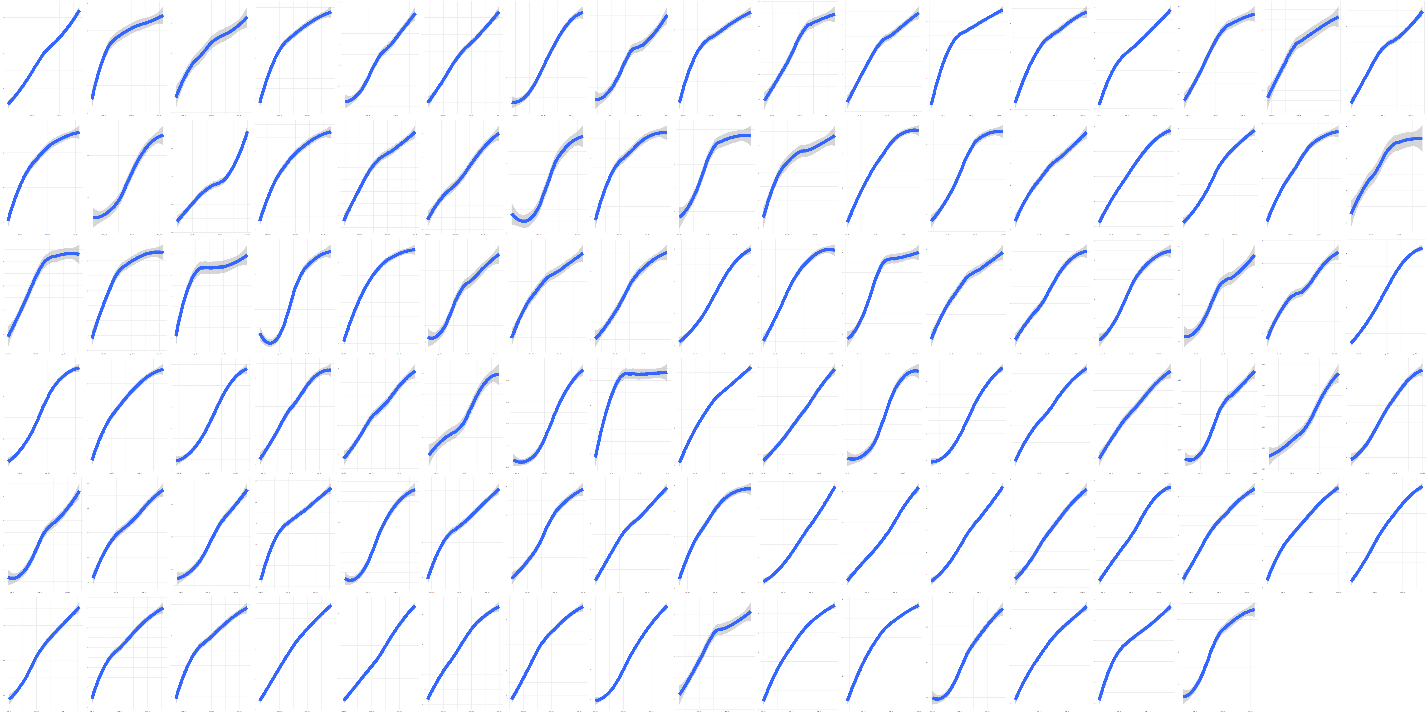

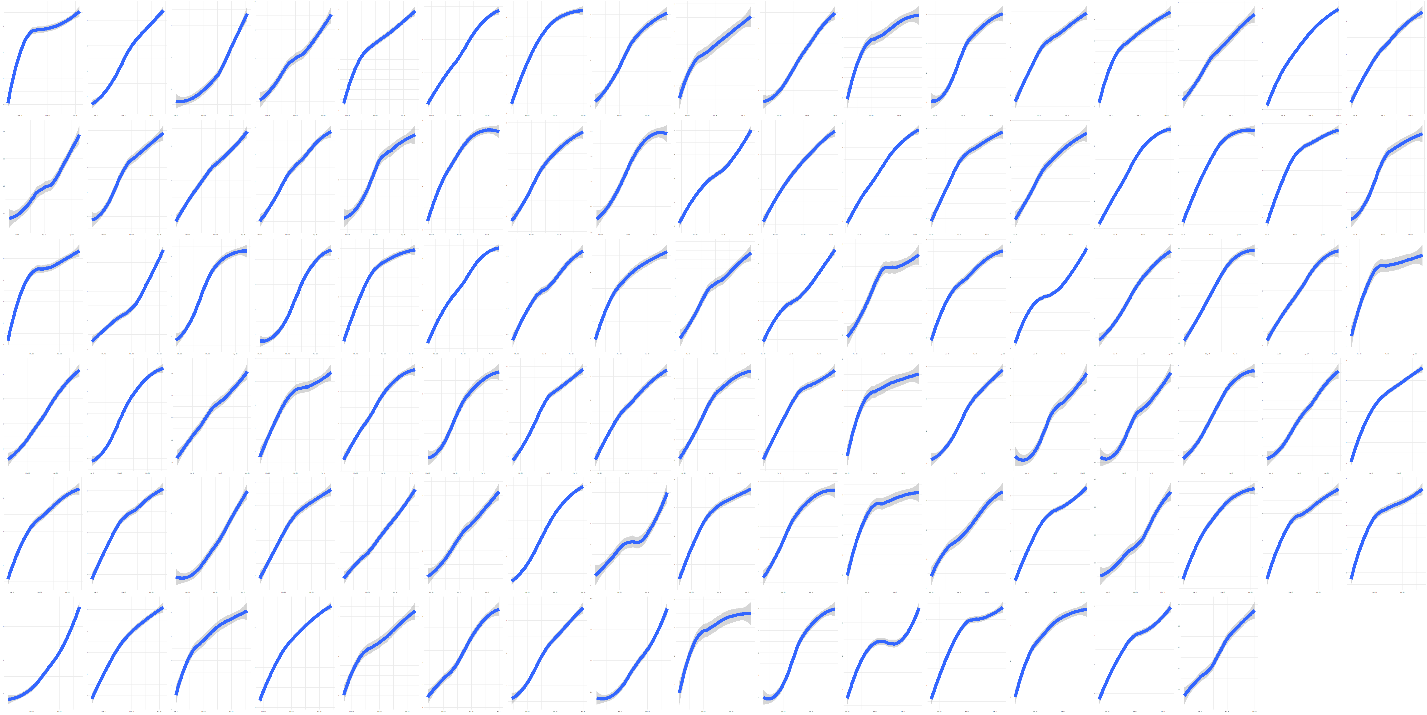

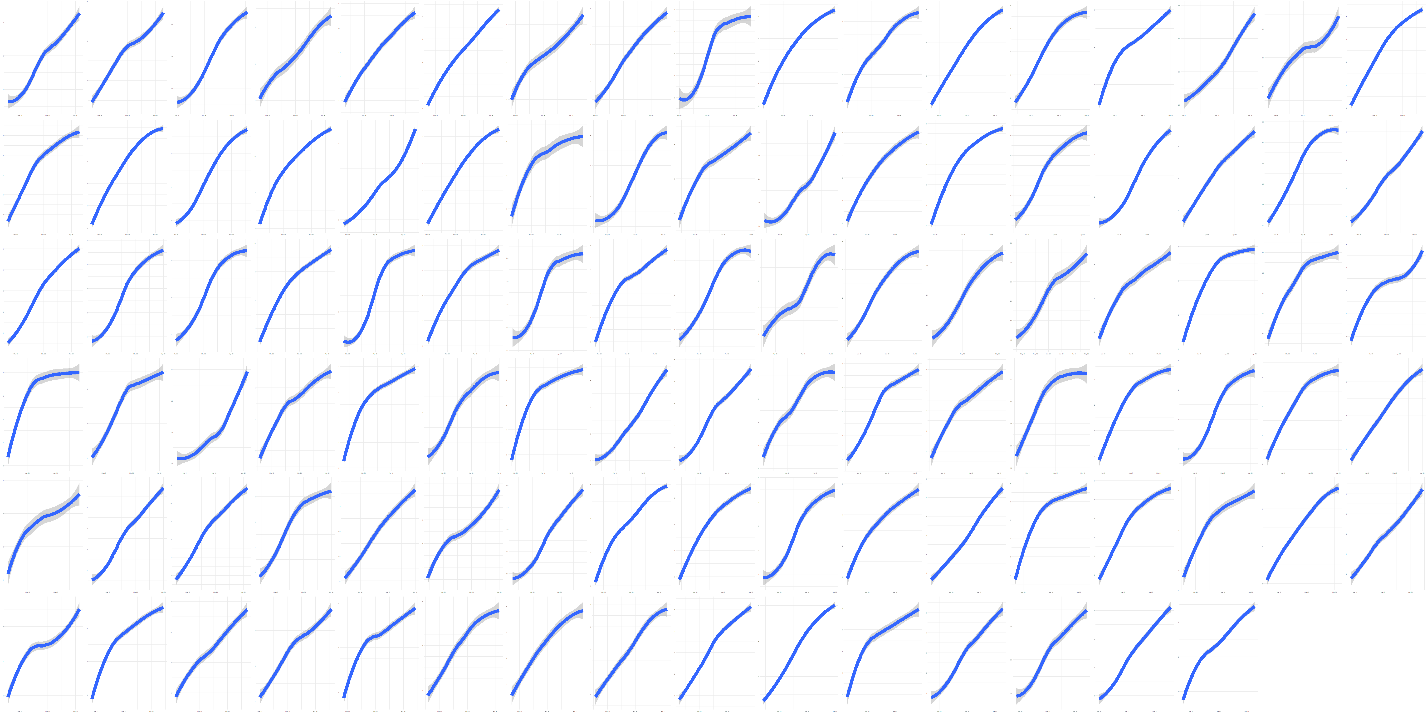

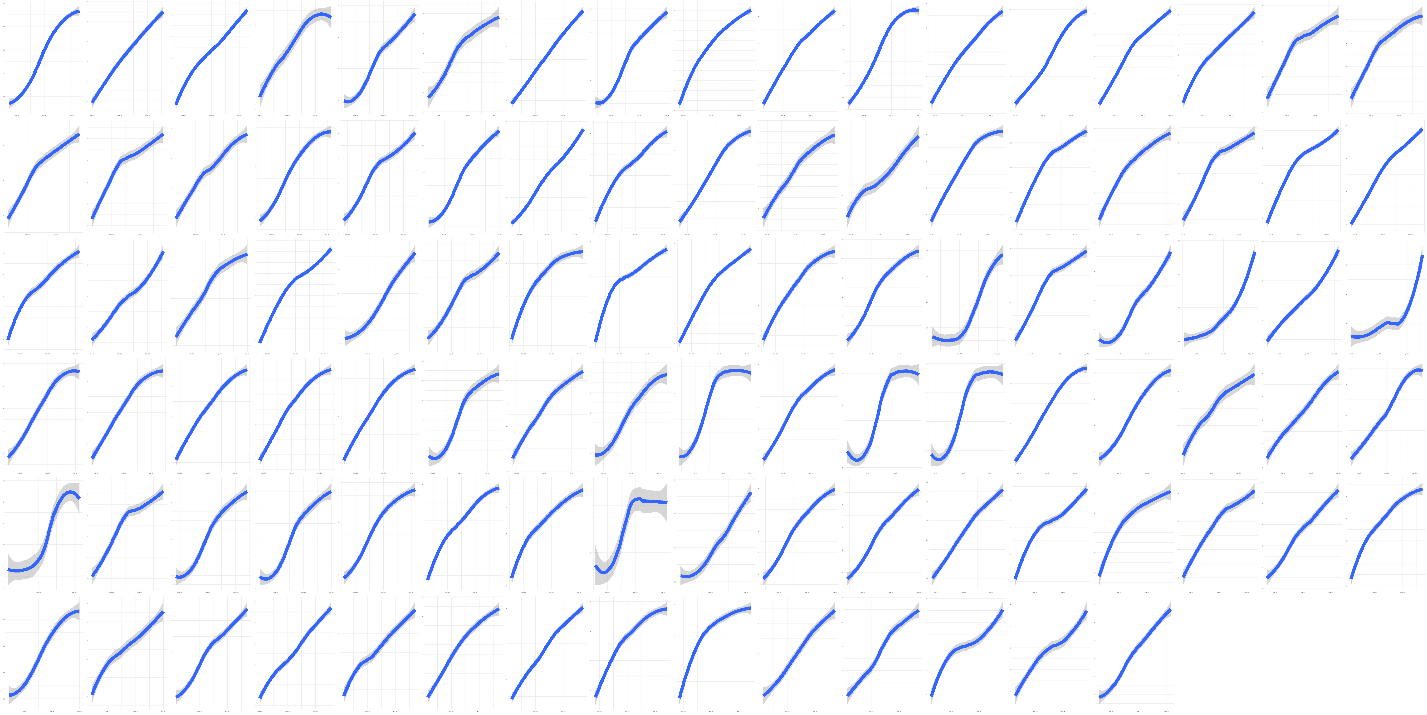

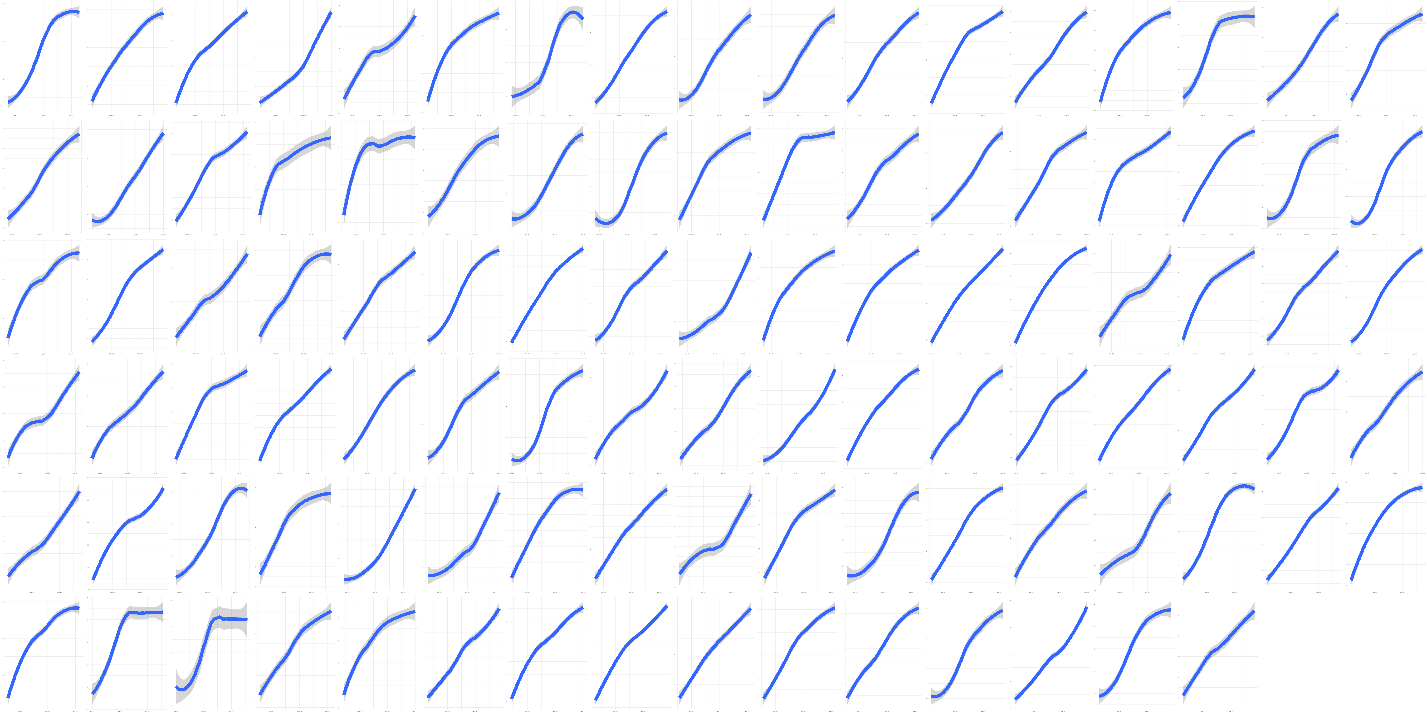


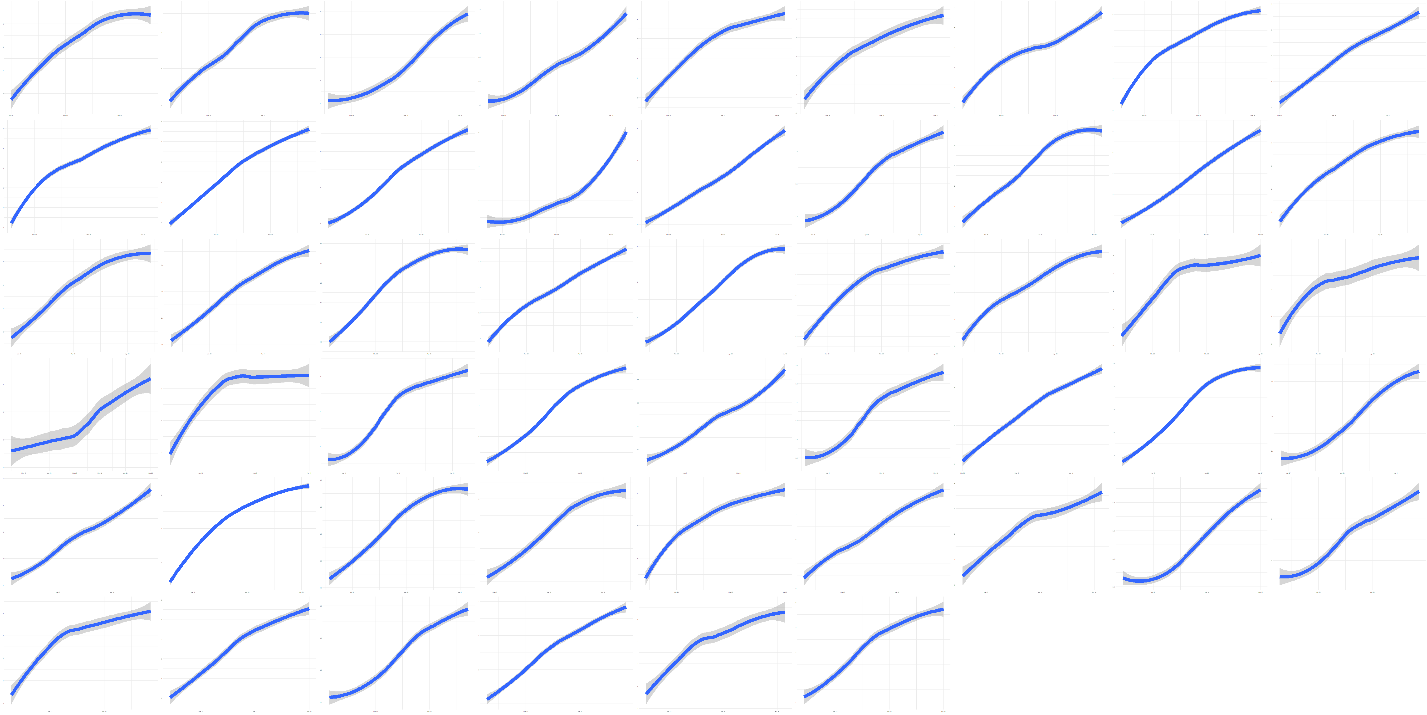


**S1 Appendix Fig B. Period of calibrated exponential growth of deaths in US counties (n=301).** The plots of the log of cumulative deaths over time for each county demonstrated reasonable curves that approximated exponential growth. The calibrated exponential growth period was used to estimate R_0_.


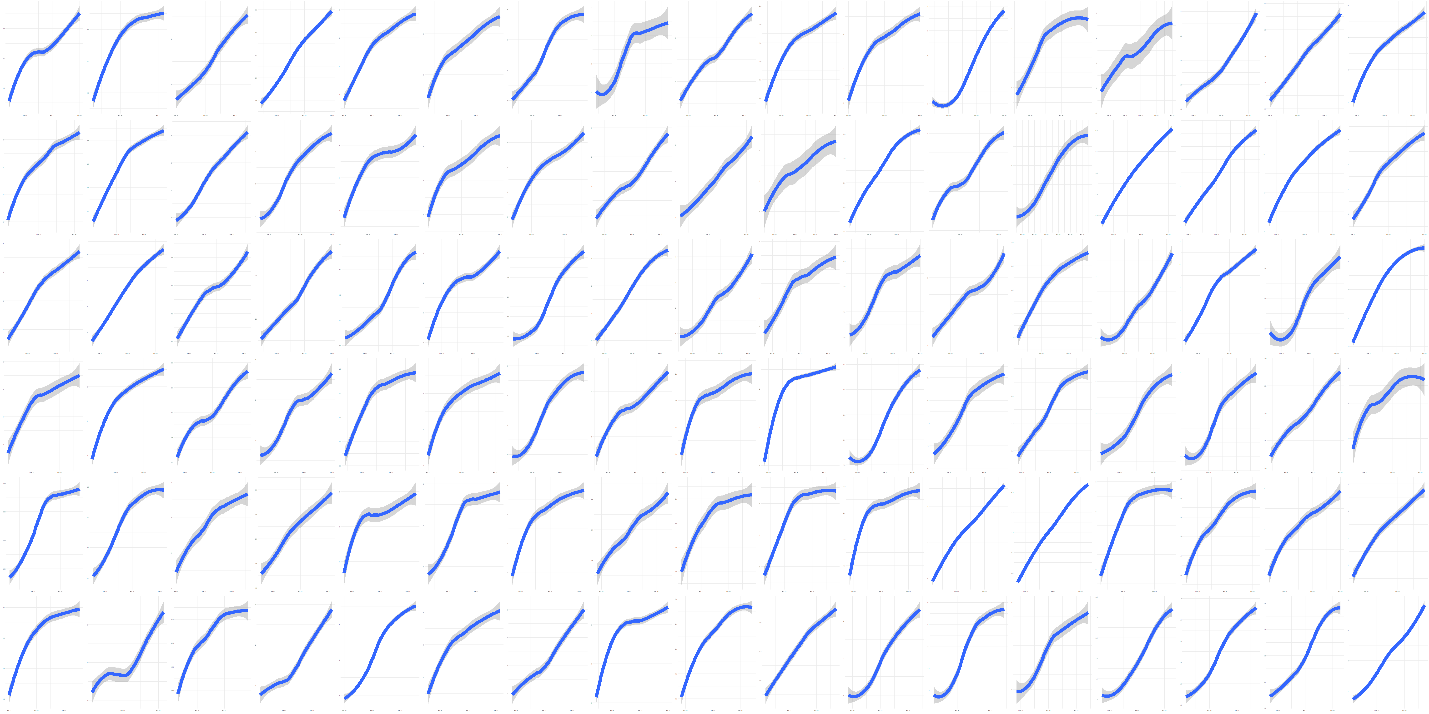

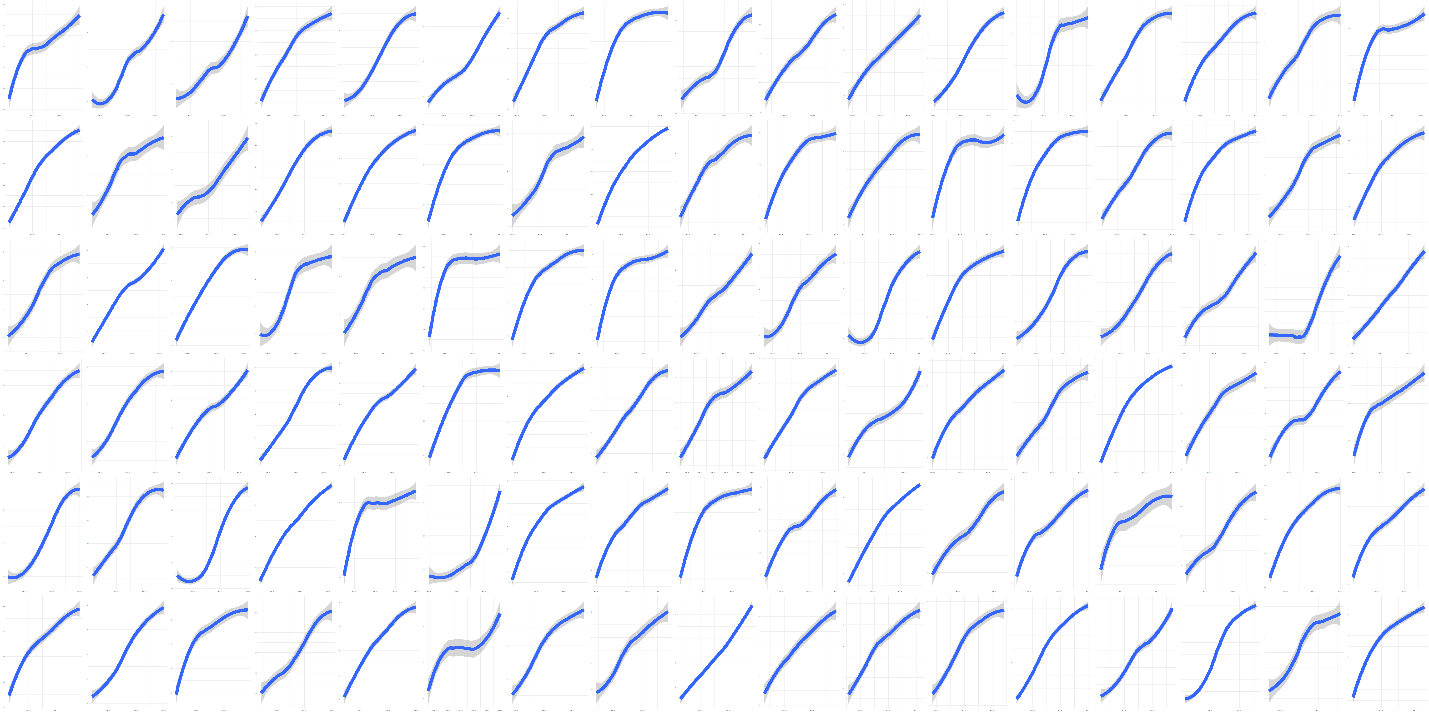


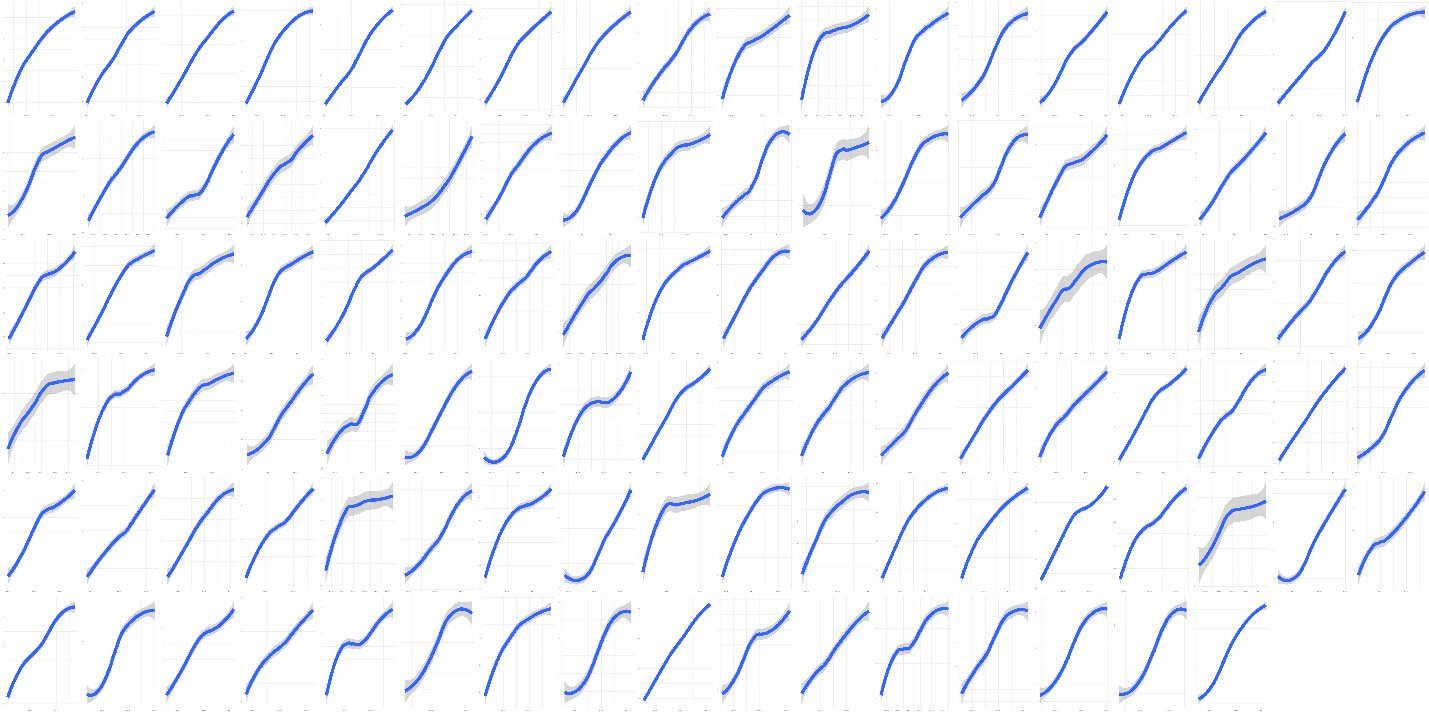

Supplement: S1 Appendix — (DOCX) [file pone.0249271.s001.docx]
